# Impact of hearing aid technology level at first-fit on self-reported outcomes in patients with presbycusis: a randomized controlled trial

**DOI:** 10.3389/fragi.2023.1158272

**Published:** 2023-06-01

**Authors:** Sabina Storbjerg Houmøller, Anne Wolff, Li-Tang Tsai, Sreeram Kaithali Narayanan, Dan Dupont Hougaard, Michael Lyhne Gaihede, Tobias Neher, Christian Godballe, Jesper Hvass Schmidt

**Affiliations:** ^1^ Research Unit for ORL—Head and Neck Surgery and Audiology, Odense University Hospital, University of Southern Denmark, Odense, Denmark; ^2^ OPEN, Odense Patient data Explorative Network, Odense University Hospital, Odense, Denmark; ^3^ Department of Otolaryngology, Head and Neck Surgery and Audiology, Aalborg University Hospital, Aalborg, Denmark; ^4^ Department of Electronic Systems, Aalborg University, Aalborg, Denmark; ^5^ Department of Clinical Medicine, Aalborg University, Aalborg, Denmark

**Keywords:** presbycusis, hearing aids, technology level, self-reported outcomes, real-ear measurements

## Abstract

To provide clinical guidance in hearing aid prescription for older adults with presbycusis, we investigated differences in self-reported hearing abilities and hearing aid effectiveness for premium or basic hearing aid users. Secondly, as an explorative analysis, we investigated if differences in gain prescription verified with real-ear measurements explain differences in self-reported outcomes. The study was designed as a randomized controlled trial in which the patients were blinded towards the purpose of the study. In total, 190 first-time hearing aid users (>60 years of age) with symmetric bilateral presbycusis were fitted with either a premium or basic hearing aid. The randomization was stratified on age, sex, and word recognition score. Two outcome questionnaires were distributed: the International Outcome Inventory for Hearing Aids (IOI-HA) and the short form of the Speech, Spatial, and Qualities of Hearing Scale (SSQ-12). In addition, insertion gains were calculated from real-ear measurements at first-fit for all fitted hearing aids. Premium hearing aid users reported 0.7 (95%CI: 0.2; 1.1) scale points higher total SSQ-12 score per item and 0.8 (95%CI: 0.2; 1.4) scale points higher speech score per item, as well as 0.6 (95%CI: 0.2; 1.1) scale points higher qualities score compared to basic-feature hearing aid users. No significant differences in reported hearing aid effectiveness were found using the IOI-HA. Differences in the prescribed gain at 1 and 2 kHz were observed between premium and basic hearing aids within each company. Premium-feature devices yielded slightly better self-reported hearing abilities than basic-feature devices, but a statistically significant difference was only found in three out of seven outcome variables, and the effect was small. The generalizability of the study is limited to community-dwelling older adults with presbycusis. Thus, further research is needed for understanding the potential effects of hearing aid technology for other populations. Hearing care providers should continue to insist on research to support the choice of more costly premium technologies when prescribing hearing aids for older adults with presbycusis.

**Clinical Trial Registration:** https://register.clinicaltrials.gov/, identifier NCT04539847.

## 1 Introduction

Hearing loss is one of the most common chronic health conditions today (Stevens et al., 2013; Besser et al., 2018; World Health Organization, 2021). According to the Global Burden of Disease studies, hearing loss is the third leading cause of disability worldwide (GBD Hearing Loss Collaborators, 2021). Age-related hearing loss (presbycusis) is projected to be one of the top 10 leading causes of burden of disease by 2030, following a global demographic shift towards an aging population (Mathers and Loncar, 2006; Davis et al., 2016). It is estimated that approximately 50 percent of people older than 60 years and 80 percent of those older than 85 years have a hearing deficit (Cunningham and Tucci, 2017), and that 30% of men and 20% of women in Europe have a pure-tone average hearing loss across 0.5, 1, 2, and 4 kHz (PTA-4) of 30 dB hearing level (HL) or more in the better ear by the age 70 years (Roth, 2011).

Presbycusis is described as the cumulative effect of aging resulting from a degeneration of the cochlea and characterized by reduced hearing sensitivity and speech understanding in noisy environments (Working Group on Speech Understanding and Aging, 1988; Gates and Mills, 2005; Cunningham and Tucci, 2017). The audiometric profile is a bilateral symmetrical sensorineural hearing loss (ISO:7029, 2017) that progresses over the years, especially in the high-frequency region (Davis et al., 2016). Due to complex genetics and environmental factors that affect hearing throughout the entire lifespan, the underlying pathology is complex and contributes to an extensive variation in audiometric profiles (Dubno et al., 2013).

Hearing aids (HA) are the conventional choice of rehabilitation for older adults with presbycusis (Kochkin, 2009; Burton, Adams and Rosenfeld, 2014; Maidment et al., 2016), and the technology has improved rapidly over the last few decades (Edwards, 2007). The most substantial change was the transition from analog to digital sound processing, allowing for more advanced signal processing strategies (Levitt, 2007; Bertoli, 2010). Hearing aids can be more or less advanced in terms of feature settings and speech processing, but essentially, they all consist of four basic blocks: a microphone, a signal processor, a loudspeaker, and a power source (Levitt, 2007; Dillon, 2013). Manufacturers produce HA families that include different models at different levels of technology. The more advanced the technology level is, the higher the cost of the HAs will generally be (Chung, 2004). When fitting patients with HAs in clinics today, the choice of HA technology level is one of the challenges clinicians encounter (Walden et al., 2000; Cox, 2014; Johnson, 2016). The decision is often based on the clinician’s individual preferences and the patient´s hearing needs. Cost is an important decision factor when total HA reimbursement is not provided. Studies have shown that patients perform a cost-benefit analysis to decide if the HAs provide sufficient value to justify their expenses (Newman, 1998; Cox, 2014). Devices that provide more benefit for a given cost are considered to provide greater value. Thus, cost has also been identified as a contributing factor for low HA uptake (Chien and Lin, 2012).

There is a lack of knowledge regarding what level of technology should be recommended for patients with a hearing loss, and further research is needed to clarify the relative benefits of premium-level *versus* basic-level HA technologies. From the HA users’ point of view, it is important to know whether there is evidence suggesting greater benefit with premium compared to basic HA technology. In other words, in the decision process it is important to know, if a premium-level HA is worth the cost, which is a highly relevant topic from both a clinical and a commercial perspective.

Real-ear insertion gain can be used to verify the actual gain provided by the HA and is defined as the sound pressure level (SPL) near the eardrum when aided, minus the SPL at the eardrum when unaided (Ching and Dillon, 2003). Variations in the individual outer ear cause a mismatch between the predicted insertion gain and the measured real-ear insertion gain (Bell, 2009), and research has shown that different gain levels are prescribed for the same type of hearing loss depending on the device model and manufacturer (Keidser, 2003; Sanders et al., 2015; Sanchez-Lopez et al., 2021). Therefore, evidence suggests that it is important to use insertion gain when fitting HAs because the first-fit of HAs cannot be relied on to provide an accurate fit (Aazh and Moore, 2007; Aazh, 2012), and guidelines from professional organizations are available to guide matching the insertion gain to target (e.g., British Society of Audiology [BSA], 2007). The number of compression channels in the HA, the option for modifications of the HA and the acoustics of the unoccluded or occluded ear canal are determining the closeness between the target and insertion gain (Bell, 2009). Research has shown that fittings made according to a verified target prescription improve speech intelligibility in quiet and noise, and that real-ear measurement (REM)-based fittings improve the self-reported HA outcomes (Moore, 2001; Ching et al., 2010; Abrams et al., 2012; Almufarrij, 2021). In premium-level HAs, the more advanced signal-processing features and greater number of compression channels, noise reduction, feedback reduction, and microphone systems, are designed to improve speech-understanding compared to more basic levels of technology (Chung, 2004; Edwards, 2007). More complex technology such as environmental adaptation and binaural data streaming are often included in premium HA models (Levitt, 2007). Premium technologies have been suggested to yield improved access to speech cues, thus reducing the attentiveness required for speech understanding and thereby decreasing listening effort (Hornsby, 2013; Johnson, 2016). Some studies have shown performance advantages in laboratory tests with modern HA technology compared to basic technology (Walden et al., 2000; Wood and Lutman, 2004; Kießling and Kreikemeier, 2013; Wu et al., 2019).

Clinical assessments of HA performance are often not predictive of real-world outcomes which underlines the importance of assessing the perceived outcome of the patients. Real-world outcomes of a HA fitting can be assessed using different methods, and one reasonable way would be to ask for the HA user´s opinion. Thus, the patient’s perspective has been argued to be the gold standard to assess HA effectiveness (Cox, 2014; Cox, 2016). Over the years, several instruments for measuring real-world outcomes have been developed, but only a limited number of these instruments are translated to Danish. The International Outcome Inventory for Hearing Aids (IOI-HA) (Cox et al., 2000; Jespersen, 2014) and the short version of the Speech, Spatial and Qualities of Hearing Scale (SSQ-12) (Gatehouse and Noble, 2004; Jensen, 2009) are among the few translated and validated Danish questionnaires. The IOI-HA measures the perceived HA effectiveness and comprises seven items, targeting different outcome domains (Cox and Alexander, 2002), whereas the SSQ addresses perceived hearing abilities in three domains (speech, spatial, and qualities of hearing) and originally entails 49 questions. An abbreviated version, SSQ-12, was developed to encourage implementation into routine clinical practice (Noble et al., 2013).

Previous research has demonstrated limited differences between premium-level and basic-level HA technologies using self-reported outcome measures. Walden et al. (2000) compared differences in perceived benefit between more advanced digital HAs and basic linear technology using the Profile of Hearing Aid Benefit outcome questionnaire and found that the participants did not perceive the performance advantages shown in laboratory testing. Cox and colleagues investigated differences in the effectiveness of premium *versus* basic HAs among 25 and 45 older individuals (mean age 70 years), respectively, with mild-to-moderate bilateral hearing loss and included both first-time and experienced HA users (Cox, 2014; Cox, 2016; Johnson, 2016; Johnson, 2017). They used both subjective and objective outcome measures to explore technology differences and included laboratory speech understanding and sound localization tests, along with four standardized questionnaires (the Abbreviated Profile of Hearing Aid Benefit (APHAB), the SSQ-B version, the Device-Oriented Subjective Outcome (DOSO), and Hearing-Related Quality of Life questionnaire). Listening effort outcomes, assessed using both laboratory tests and subjective ratings, showed no significant differences between the technology levels (Johnson, 2016), neither did the self-reported outcome measures (Cox, 2014; Cox, 2016). Wu et al. (2019) investigated technology differences related to directional microphones and noise reduction and found that premium HAs outperformed basic HAs in laboratory setting, but this was not apparent in real-world. The findings of a more recent study by Plyler et al. (2021) are consistent with Cox and colleagues, but they found that noise acceptance and satisfaction for speech in larger groups were significantly improved with premium devices and that those in more demanding listening environment received significant improvements with premium HAs.

The current study contributes to further research on the efficacy of premium-feature devices compared to basic-feature devices. The study was conducted as a randomized controlled trial to strengthen the results, which provided a homogenous patient group and minimized patient related variation. To provide clinical guidance in HA prescription, we aimed to investigate if arguments for prescribing a more costly premium HA for older adults with presbycusis could be found in a clinical set-up where HAs are costless for the patients. Thus, the main purpose of the study was to test the hypothesis that premium technologies provide better self-reported hearing abilities and greater perceived HA effectiveness compared to basic technologies among older adults with symmetric presbycusis. Secondly, as an explorative analysis, we investigated if differences in gain prescription between the six chosen HA models as verified with REM could explain differences in reported outcomes between premium-level and basic-level HA users.

## 2 Materials and methods

### 2.1 Study design and ethics

The study was designed as a two-arm parallel randomized controlled trial. Data were collected at the Odense site as part of the Danish national Better-hEAring-Rehabilitation (BEAR) project that aims to improve audiological rehabilitation in Denmark and worldwide through an evidence-based renewal of clinical practice. Data in the BEAR study were collected from the Department of Audiology at Odense University Hospital (OUH), Region of Southern Denmark and the Department of Audiology at Aalborg University Hospital (AAUH), North Denmark Region from January 2017 to May 2018. Adults (≥18 years of age) referred for public HA treatment were enrolled in the BEAR study, regardless of previous HA experience. The BEAR project was evaluated by The Regional Committees on Health Research Ethics for Southern Denmark (S-20162000-64), and the present study was registered (Clinical trial registration NCT04539847).

### 2.2 Population and procedure

Data were collected from 1,159 patients with hearing loss who were a subgroup of patients enrolled from Region Southern Denmark accepting to participate in the BEAR project (mean age 68 ± 12 years, 45% women). These patients were distributed across the whole BEAR study period. They were recruited by private ear-, nose-, and throat (ENT) physicians that were informed about the study, and project information letters were sent to the ENTs to distribute to the patients. All patients received a letter including information about study details, a consent form, and a note on the patient´s rights related to study participation. The consent to participate was forwarded in a referral letter to the Department of Audiology, OUH. The inclusion criteria for the current study were patients with bilateral presbycusis with no previous experience with HAs. Presbycusis was defined as a symmetrical hearing loss (less than 10 dB difference in PTA-4 between right and left ear) in patients older than 60 years where the high-frequency hearing loss were greater than the low-frequency hearing loss, and no disclosed history of hearing loss besides age. Patients were excluded if they were not native Danish speakers, not able to complete the consent form or the questionnaires, or if they were experienced HA users. Each patient’s audiogram and general medical history was contained in the referral letter from the private ENT specialists and checked by the responsible researcher. If the inclusion criteria were met, patients were randomly assigned to either a premium-feature or basic-feature HA, stratified on age (60–69 years, 70–79 years, 80 years or above), sex, and word recognition scores (WRS) (WRS≥80% and WRS<80%). Block randomization with varying block sizes (12 and 18) was managed in the electronic REDCap database enabling equal distribution of the stratification variables in the two groups. Allocation was concealed.

**FIGURE 1:**
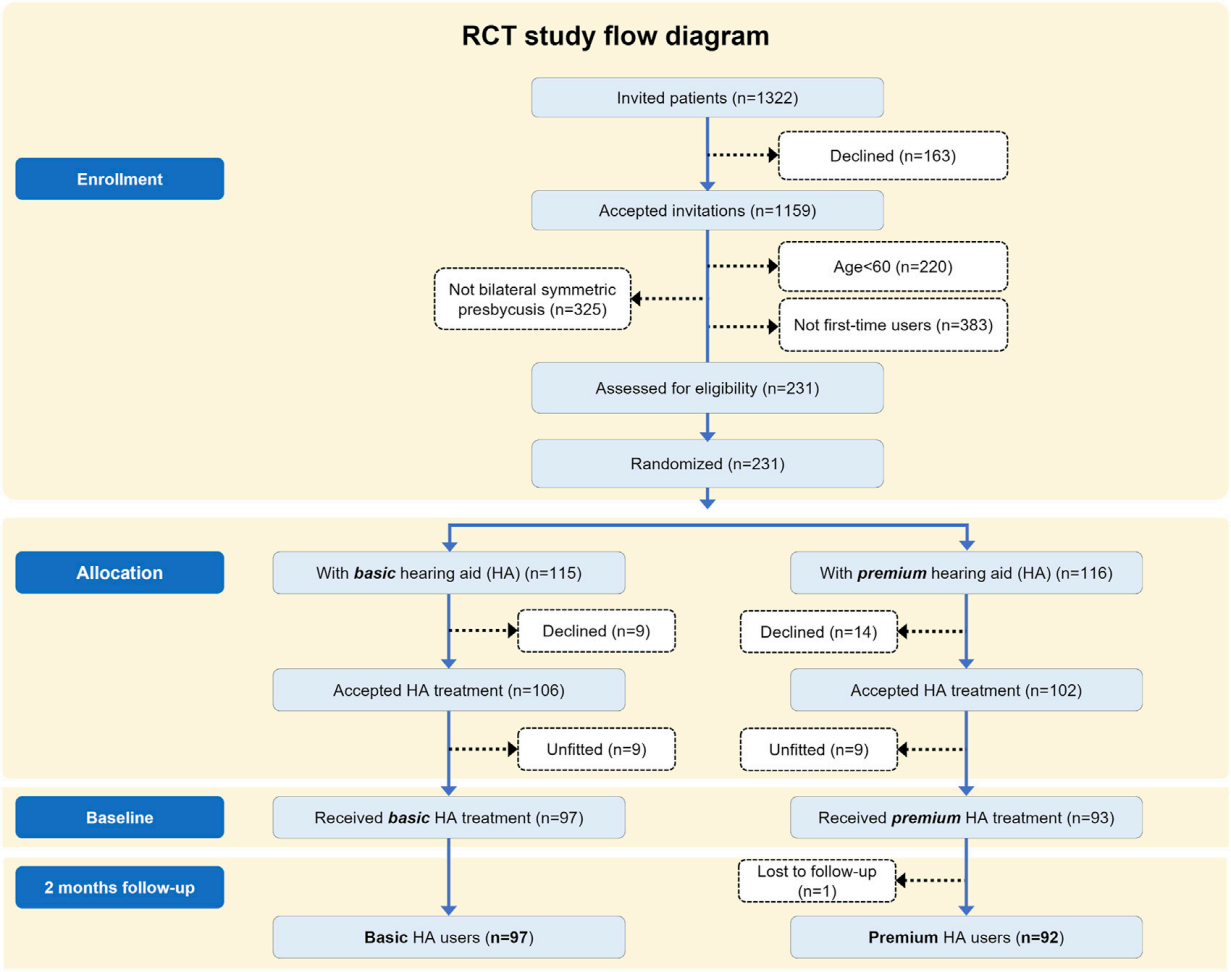
Patient flow diagram.

In total, 231 patients were eligible for the study and randomized into two different groups (*n* = 115/116) (Figure 1). Twenty-three patients declined the HA treatment after given consent to participate, resulting in a study population of 208 patients. One hundred and six patients were allocated to basic-feature HAs, and 102 patients were allocated to premium-feature HAs. Patients were only presented to the randomly assigned HA model, but the audiologist could select another HA, or the patient could decline treatment with the selected HAs. Eighteen patients were fitted with HAs other than the allocated model and therefore excluded, leaving a study population of 190 patients (97 basic HA users and 93 premium HA users) (Figure 1). Patients were informed that the research was about improving hearing rehabilitation, but they were blinded towards the random selection of HAs and about taking part in the randomized study. No special attention was given towards this randomized subgroup within the BEAR project with regards to the HA fitting and counselling process, and the researchers were not actively involved in the intervention to promote the study. Patients were informed of the standard 2-months trial period with the option of choosing another HA model, or discontinue the HA treatment, if they were dissatisfied with the fitted HAs. The name of the individual HA manufacturer was visible to the patients, and patients therefore had the possibility to obtain information on the level of technology, but the potential cost or level of technology of the HAs were not discussed.

### 2.3 Hearing aids

Six different pairs of commercially available HAs (Resound Enya 4, Resound LiNX2 7, Oticon Nera2 Pro, Oticon Opn 1, Widex Dream 220 Fusion, and Widex Unique 440 Fusion) were evaluated as examples of basic-feature and premium-feature technologies from three manufacturers contributing to the BEAR project. One pair of basic-feature and premium-feature HA was included from each manufacturer. The HAs were released in different years and used different platforms, and the fitting strategy in one of the three companies differed between the two levels of HA technology, which is reported in Smeds et al. (2016). The HA models are anonymized in the following sections to comply with the collaboration agreement of the BEAR project. In Denmark, it is possible to receive free HAs as part of the public healthcare system, and therefore, patients can be treated with selected types of HAs that have been included as part of a regular ongoing public tender. All selected HAs in this study were available for the public tender in Denmark, and thus, representative of the HAs accessible for patients receiving their HAs free of charge from the Danish public healthcare system. A balanced design was applied with an intended representation of approximately one-third for each of the three manufacturers to avoid the dominance of a specific HA product and accompanying fitting rationale. To ensure equal distribution of the HAs from the three manufacturers, a randomization tool was used. The six different HA models were all behind-the-ear devices corresponding to the most popular style currently marketed. A list of the advertised features in each model of HA is presented in Table 1. The three premium HAs contained more compression channels and more advanced processing features (e.g. adaptive microphone directionality, noise management including wind noise reduction, environmental adaptation, and proprietary high-frequency boost). Besides, the three premium models included connectivity which enabled the use of HAs with smartphones.

**TABLE 1:** Differences between premium- and basic-features in the six hearing aids as described by manufacturers (A, B, and C).

Features	Hearing aids					
	Premium A	Basic A	Premium B	Basic B	Premium C	Basic C
Number of compression channels	16	8	15	5	17	10
Adaptive microphone directionality	More advanced	Less advanced	More advanced	Less advanced	More advanced	Less advanced
Pinna simulation	N/A	N/A	Yes	No	Yes	No
Noise management	More advanced	Less advanced	Yes	No	More advanced	Less advanced
Wind Noise reduction	Yes	No	Yes	No	More advanced	Less advanced
Feedback management	Yes	Yes	Yes	Yes	Yes	Yes
Environmental adaptation	Yes	No	Yes	No	Yes	No
Binaural data streaming	Yes	Yes	Yes	No	Yes	Yes
Proprietary high-frequency boost	More advanced	Less advanced	Yes	No	Yes	Yes
Sound quality enhancer	Yes	No	Yes	No	Yes	No
Speech enhancer	More advanced	Less advanced	Yes	No	N/A	N/A
Number of programs	4	4	5	3	4	4
Tinnitus support	Yes	Yes	Yes	No	Yes	Yes
Connectivity (iPhone, Android)	Yes	No	Yes	No	Yes	No

Not applicable, N/A, was applied if the feature was not relevant for the given HA model, or if no information was available on the specific feature

### 2.4 Hearing aid fittings

All patients were bilaterally HA fitted by an experienced audiologist according to standard clinical practice, and the HAs were linked to the fitting software with wireless communication. The fitting and fine tuning of HAs were carried out in a single session following hearing evaluation. HAs were fitted using the proprietary fitting rationales by the specific HA manufacturers with NAL-NL2 target gains only being used for reference purposes in the analysis. Based on feedback from patients, If necessary, some gain adjustments in the high-frequencies were carried out to achieve a fit acceptable to the HA user. The decision about the acoustic coupling was based on individual characteristics of the ear canal and recommendations in the fitting software. Table 2 provides an overview of the final choice of acoustic earpiece per level of HA technology.

**TABLE 2:** Distribution of the different types of acoustic fit (*n* = 190) by level of hearing aid technology.

Acoustic fit	Frequency	
	Premium	Basic
Open dome	58	57
Closed dome	14	7
Tulip dome (semi-open)	9	7
Custom made (silhouette)	5	9
Micro mold	4	7
Double Tulip (power)	1	2
Casted mould (shell)	0	1
N/A	2	7
Total	**93**	**97**

Total number of fitted hearing aids marked in bold.

All feature settings were set according to the recommendations of the manufacturer and therefore left in default setting. As all patients were first-time HA users, no additional programs were added to the default listening program, and patients were given a short instruction on how to use the HAs, clean the earmolds, and changing of batteries. If connectivity was available for the given HA model, the HAs were connected via Bluetooth. Patients in need of assistive listening devices (e.g. remote controls) were referred to the responsible agencies or personnel. They were all recommended to use the devices during waking hours for as long as possible. Besides, they were informed of the opportunity to ask for additional counselling if needed.

### 2.5 Measures

#### 2.5.1 Questionnaires

All patients completed a questionnaire survey 2 weeks before the first visit to the clinic and 2 months after HA fitting. The outcomes were designed to capture the perceived hearing abilities and the effectiveness of HAs and included the SSQ-12 (Gatehouse & Noble, 2004; Noble et al., 2013) and the IOI-HA (R. Cox et al., 2000). As all patients were first-time users, they only responded the IOI-HA 2 months following HA fitting. The SSQ was designed to assess people’s perception of their listening capabilities in various situations and consists of the three domains: speech, space, and sound quality. In the SSQ-12 version, the three domains are represented by fewer questions (speech domain: 5 questions, space: 3 questions, sound quality: 4 questions) compared to the original version that entails 49 questions. The scale is ordinal and ranges from 0 to 10. A higher score reflects better hearing ability. The IOI-HA is a seven-item questionnaire intended to probe the experience with HAs during the recent past (2 weeks), reflecting the overall HA effectiveness. The scale is ordinal and ranges from 1 to 5. A higher score indicates a better outcome in the specific domains. Using principal component analysis or factor analysis, previous studies have identified two subscales within the IOI-HA that is described as factor 1 and factor 2 scores and reflect two different aspects: the HA benefit and the remaining difficulties with HAs (Cox and Alexander, 2002; Kramer et al., 2002; Brännström and Wennerström 2010; Jespersen et al., 2014). A non-standardized health-related questionnaire was also included in the baseline questionnaire survey and contained questions on demographical details such as sex, age, occupational status, HA experience, and motivation. The patients’ motivation for HA treatment was assessed by two questions from an online evidence-based motivation tool developed by the Ida Institute (idainstitute.com): ‘How important is it for you to improve your hearing?’ and ‘How much do you believe in your ability to use hearing aids?’. The scale is ordinal and ranges from 0 to 10. A higher score indicates higher motivation (Clark, 2010).

The questionnaire survey was managed by the research electronic data capture (REDCap) software that was developed by Vanderbilt University, Nashville, Tennessee, United States and hosted by Odense patient explorative network (OPEN) in the Region of Southern Denmark (Harris et al., 2009; 2019). All patients received and answered the questionnaire survey through an online link generated by REDCap. Due to the online versions of the questionnaires, some modifications were made to the SSQ-12. The response scale from 0–10 was marked by a cursor placed at the point of the scale corresponding to one’s specified score. The response option, ‘not applicable,’ was substituted with an option to leave the cursor untouched, corresponding to the answer, ‘not applicable,’ or ‘do not know.’ A paper version was also available at the clinic for patients who were unable to fill out the form electronically. The responses were manually entered into the database by a research assistant.

#### 2.5.2 Audiological assessment

As part of the first visit to the clinic, all patients underwent standard audiometry according to current clinical practice. The audiological assessment included a pure-tone audiometry measuring air-conduction thresholds for left and right ears at 250 Hz, 500 Hz, 1 kHz, 2 kHz (3 kHz), 4 kHz (6 kHz) and 8 kHz; bone-conduction thresholds at 250 Hz to 4 kHz when air-conduction thresholds showed low-frequency hearing thresholds >20 dB hearing lossor were asymmetric between the two ears; and a measure of WRS. Tympanometry was measured to rule out any middle-ear diseases. Air- and bone-conduction thresholds were measured according to ISO8253-1:2010 (International Organization for Standardization). TDH39 headphones, or ER-3A insert earphones, were used during the tests. The WRS was obtained by presenting 25 different monosyllabic words in quiet at the most comfortable listening level from the validated DANTALE-I wordlists (Elberling et al., 1989). The result is expressed as the percentage of correct responses to the words presented. All measurements were conducted in a soundproof booth in the Audiological Department at OUH and were carried out by experienced audiologists.

#### 2.5.3 Real-ear measurements

A follow-up appointment was scheduled approximately 2 months after HA fitting. Real-ear measurements were performed both before and after any adjustments of the HAs and included measuring real-ear unaided gain at 65 dB SPL to record the natural gain provided by the outer ear followed by measuring real-ear aided gain at three different input levels (55, 65, and 80 dB SPL). Finally, the real-ear insertion gain was derived by subtracting the unaided gain from the aided gain. Only the REMs that were obtained before any adjustments of the HAs were used in the analysis. The International Speech Test Signal (ISTS) was used as the stimulus, and the HAs were set to default so that all features were activated during the measurement. The REM module (REM440) of Affinity 2.0 (Interacoustics) was used and followed the standards: ANSI/ASA S3.46 (2013); IEC 61669 (2015); ISO 12124 (2001). Calibration of the REM headset was repeated before each session. The REMs were only used for documentation and not as a basis for adjusting the HAs. Hence, the NAL-NL2 fitting prescription was only used as a hypothetical reference target. In addition, the HA usage time in hours per day was extracted from the fitting software. Some of the logged data showed usage time of >18 h per day. This could have been due to patients not turning off the HAs during the night, and these data were therefore excluded. The follow-up visit was carried out by the two researchers at the Department of Audiology at OUH responsible for collecting the outcome data.

### 2.6 Statistical analysis

PTA-4 was calculated as the average of hearing thresholds at 0.5, 1, 2, and 4 kHz for each ear. Data normality was verified using Q-Q plots. Multiple linear regression (MLR) analyses were used to test the hypothesis that premium-feature HAs yielded better self-reported outcomes than basic-feature technologies in terms of overall SSQ score, SSQ domain scores, IOI-HA total score, and IOI-HA factor 1 and factor 2 scores. Baseline SSQ-12 scores were included in the models with SSQ-12 as an outcome to adjust for the unaided score, thereby minimizing the effect of individual differences that existed before HA treatment. Using linear regression as statistical model, the outcomes are treated as an interval scale despite ordinal scale properties. Since ordered logistic regression did not show any significant differences to the model estimates, linear regression was used in the analyses. Bootstrapping was applied to compensate for non-normally distributed residuals. Significance levels were set at *p <* 0.05 for the MLR analyses (command regress in STATA). Variance inflation factor (VIF) was used to test multi-collinearity between independent variables in all linear regression models and showed no indications for multi-collinearity in the models (VIF<2.5). The dependent variables used in the models were: IOI-HA total score, IOI-HA factor 1 and factor 2 score, SSQ-12 speech, SSQ-12 space, SSQ-12 sound quality score, and SSQ total score. The primary predictor used in the analyses was HA technology level, and secondary predictors were sex, age, WRS, motivation (Q1 and Q2), baseline SSQ-12 scores for the given domain, and mean gain deviation from NAL-NL2 targets at 1, 2, and 4 kHz. As an extended model, it was investigated whether the difference in manufacturer was a significant factor related to the outcome. The significant results from parametric analysis were checked and confirmed with non-parametric analysis using Mann-Whitney U tests as well.

Data management and analyses were performed using STATA SE version 16.0 (Stata Corp., College Station, TX).

#### 2.6.1 Power calculation (SSQ-12 and IOI-HA)

Given an observed standard deviation (SD) of 1.54 and 1.61 scale points for the overall SSQ-12 score in the two study arms, a sample size of n = 190 and a power = 0.80, we can detect a difference in the overall SSQ-12 of 0.64. Also, based on the SD of 4.39 and 4.94 scale points for IOI-HA total score in the two groups and with the same sample size of *n* = 190 and power = 0.80, we can detect a difference in IOI-HA total score of 1.91. Alpha was set to 0.05.

## 3 Results

### 3.1 Demographics

**FIGURE 2:**
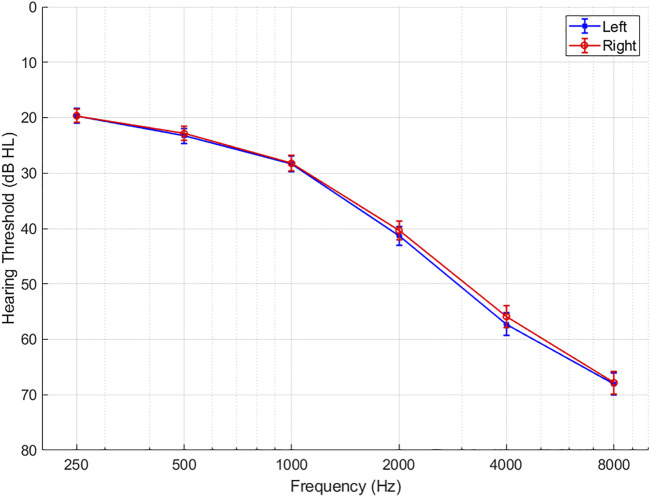
Average hearing thresholds in dB HL for left and right ear in the total study population (n = 190). Error bars show 1 standard error of the mean.

Figure 2 depicts the mean audiograms of the right and left ear for the 190 included patients. Table 3 describes the characteristics of the patients allocated to either premium or basic level technologies (*n* = 97/93). The median age of the two groups of HA users was 72 (interquartile range (IQR) 10) years and 70 (10) years, respectively. Forty-one to 45% were female HA users. The median WRS for the left and right ears of the two groups of HA users were 92% and the IQRs were 8% and 12%, respectively. The average PTA-4 levels for the left and right ears were 37.5 (12.5) dB HL and 36.4 (11.0) dB HL, respectively. The two groups were highly similar in terms of sex (*p* = 0.8), age (*p* = 0.2), PTA-4 right and left ear (*p* = 0.5, *p* = 0.5), WRS right and left ear (*p* = 0.9, *p* = 0.7), level of motivation Q1 and Q2 (*p* = 0.9, *p* = 0.4), and occupational status (*p* = 0.5). Also, there was no statistically significant difference in how much they used their HAs in hours per day in the two groups (*p* = 0.9).

**TABLE 3:** Characteristics of patients allocated to premium- or basic-feature hearing aids (HA).

Median (IQR)	Basic HA users (N = 97)	Premium HA users (N = 93)	*p*-value*
Sex, Women (%)	45	41	0.8
Age, years	72.0 (10.0)	70.0 (10.0)	0.2
Range	(61–90)	(60–93)	
PTA-4, dB HL			
Right ear	37.5 (12.5)	36.3 (10.0)	0.5
Left ear	37.5 (12.5)	36.6 (12.1)	0.5
Range	(15–60)	(18–64)	
WRS, %			
Right ear	92.0 (12.0)	92.0 (8.0)	0.9
Left ear	92.0 (8.0)	92.0 (8.0)	0.7
Severity of hearing loss based on better ear PTA-4, n (%)			0.7
Normal Hearing, ≤19 dB HL	3 (3)	1 (1)	
Mild, 20–34 dB HL	43 (44)	45 (48)	
Moderate, 35–49 dB HL	45 (46)	44 (47)	
Moderate-Severe, 50–64 dB HL	6 (6)	4 (4)	
Severe & Profound, >65 dB HL	0 (0)	0 (0)	
Motivation Q1 score	7.9 (3.1)	8.1 (3.2)	0.9
(Range 0–10)	(3–10)	(2–10)
Motivation Q2 score	8.1 (3.2)	8.5 (2.6)	0.4
(Range 0–10)	(1–10)	(1–10)	
Occupational status, n (%)			
Active	9 (9)	12 (13)	0.5
Retired	81 (84)	75 (81)	
Missing	7 (7)	6 (6)	
Average HA usage time, hours per day	9.0 (7.0)	9.0 (7.5)	0.8
Range (3–18)	(3–16)	(3–18)	
IOI-HA total score (follow-up)	29 (6)	30 (7)	0.86
Range (7–35)	(15–35)	(14–35)	
Overall follow-up SSQ-12 score (item score)	6.3	7.3	0.01**
Range (0–10)	(3–10)	(3–10)	
Speech domain (5 items)	5.6	7.0	0.01**
Spatial domain (3 items)	7.4	7.8	0.12
Qualities domain (4 items)	6.9	7.7	0.03**
Overall baseline SSQ-12 score (item score)	5.1	5.1	0.69
Range (0–10)	(3–10)	(3–10)	
Speech domain (5 items)	4.2	3.9	0.88
Spatial domain (3 items)	6.3	6.7	0.48
Qualities domain (4 items)	5.6	5.9	0.61

Hearing aids, HA; Inter-Quartile Range, IQR; Hearing level, HL; Pure-Tone Average at 0.5, 1, 2, and 4 kHz, PTA-4; Word Recognition Score, WRS. *p-values using Fisher´s exact test, Mann-Whitney *U* test, *t-*test, and Chi-Square test; **significance at *p* < 0.05

### 3.2 Self-reported outcomes using the IOI-HA and SSQ-12

Table 4 shows the results from the regression analyses that investigated if premium-feature HA users reported significantly higher overall HA effectiveness using the IOI-HA and better hearing abilities using SSQ-12 than basic-feature HA users. The results show that premium-feature HA users did not report a significantly higher IOI-HA factor 1 score (diff = 0.03, 95%CI: 1.1; 1.2, *p = 0.96*), factor 2 score (diff = -0.11, 95%CI: 0.7; 0.5, *p =* 0.73), or IOI-HA total score (diff = -0.03, 95%CI: 1.6; 1.7, *p =* 0.97) compared to basic-feature HA users. Using the SSQ-12 speech, space, and qualities domain score as outcome variables showed that premium-feature HA users reported a 0.8 (95%CI: 0.2; 1.4, *p* = 0.01) scale points higher speech score per item in the speech domain and a 0.6 (95%CI: 0.2; 1.1, *p* = 0.01) scale points higher qualities score per item in the qualities domain compared to basic-feature HA users. Using the SSQ-12 total score, results showed that premium-feature HA users reported a 0.7 (95%CI: 0.2; 1.1, *p* < 0.001) scale points higher total score than basic-feature HA users. SSQ-12 differences similarly ranged from 0.5-1 between two different noise management settings in Andersson et al. (2021) whereas the differences between the two device technology levels in Plyler et al. (2021) ranged from 0.2–0.5. Including manufacturer in the analysis did not change the significance of the results or the coefficients, hence it was eliminated from the regression model.

**TABLE 4:** Pannel (A) Associations between self-reported hearing aid (HA) outcome using the international outcome inventory for hearing aids (IOI-HA) score and level of technology. Pannel (B) Associations between self-reported hearing aid (HA) outcome using the speech, spatial, and qualities of hearing (SSQ-12) score and level of technology.

Explanatory variables	IOI-HA factor 1 (n = 157)		IOI-HA factor 2 (n = 157)		IOI-HA total (n = 157)	
	Adj. *R* ^2^ = 0.08		Adj. *R* ^2^ = 0.05		Adj. *R* ^2^ = 0.08	
	*Coef. (95% CI)*	*p-value*	*Coef. (95% CI)*	*p-value*	*Coef. (95% CI)*	*p-value*
Primary explanatory variable						
HA technology level (Ref: Basic)	0.03 (-1.15; 1.22)	0.96	-0.11 (-0.74; 0.52)	0.73	-0.03 (-1.62; 1.68)	0.92
Secondary explanatory variables						
WRS (average left and right ear)	0.01 (-0.07; 0.09)	0.82	0.03 (-0.01; 0.08)	0.14	0.04 (-0.06; 0.14)	0.47
Motivation Q1	0.28 (-0.07; 0.64)	0.12	-0.02 (-0.22; 0.18)	0.84	0.23 (-0.24; 0.7)	0.43
Motivation Q2	0.28 (-0.06; 0.61)	0.09	0.05 (-0.14; 0.24)	0.54	0.32 (-0.11; 0.77)	0.13
Sex (ref: men)	0.17 (-0.96; 1.30)	0.77	-0.22 (-0.86; 0.42)	0.50	-0.18 (-1.55; 1.45)	0.84
Age	0.00 (-0.08; 0.09)	0.94	-0.01 (-0.06; 0.03)	0.59	-0.01 (-0.12; 0.10)	0.88
REIG deviation from target at						
1 kHz	-0.09 (-0.24; 0.07)	0.32	-0.03 (-0.11; 0.06)	0.57	-0.16 (-0.32; 0.10)	0.29
2 kHz	0.07 (-0.15; 0.29)	0.56	0.07 (-0.06; 0.19)	0.29	0.03 (-0.16; 0.43)	0.86
4 kHz	0.00 (-0.15: 0.15)	0.97	0.01 (-0.08; 0.09)	0.84	0.14 (-0.19; 0.21)	0.17
Constant	10.34 (-1.33; 22.02)	0.09	11.03 (4.45; 17.61)	0.00	21.37 (5.84; 36.91)	0.01

Explanatory variables	SSQ speech (n = 158) Adj. *R* ^2^ = 0.22		SSQ spatial (n = 158) Adj. *R* ^2^ = 0.38		SSQ qualities (*n* = 157) Adj. *R* ^2^ = 0.22		SSQ total (n = 157) Adj. *R* ^2^ = 0.33	
	*Coef. (95% CI)*	*p-value*	*Coef. (95% CI)*	*p-value*	*Coef. (95% CI)*	*p-value*	*Coef. (95% CI)*	*p-value*
Primary explanatory variable								
HA technology level (ref: Basic)	0.80 (0.2; 1.4)	**0.01**	0.41 (-0.1; 0.9)	0.11	0.64 (0.2; 1.1)	**0.01**	0.67 (0.2; 1.1)	**<0.001**
Secondary explanatory variables								
WRS (average of left and right ear)	0.04 (0.0; 0.1)	0.06	0.04 (0.01; 0.1)	**0.02**	0.02 (-0.01; 0.1)	0.24	0.03 (0.0; 0.1)	**0.05**
Motivation Q1	0.19 (-0.1; 0.4)	0.08	0.21 (0.1; 0.4)	**0.03**	0.12 (-0.03; 0.3)	0.09	0.19 (0.1; 0.3)	**0.01**
Motivation Q2	0.01 (-0.2; 0.9)	0.96	0.02 (-0.1; 0.2)	0.77	0.01 (-0.1; 0.1)	0.93	0.00 (-0.1; 0.1)	0.98
Sex (ref: men)	-0.36 (0.9; 0.2)	0.22	-0.74 (-1.3;-0.2)	**0.01**	-0.09 (-0.6; 0.4)	0.71	-0.35 (-0.8; 0.1)	0.12
Age	-0.04 (-0.1; 0.0)	0.06	-0.01 (-0.1; 0.03)	0.59	-0.02 (-0.1; 0.01)	0.22	-0.02 (-0.1; 0.01)	0.19
REIG deviation from target at								
1 kHz	-0.02 (-0.1; 0.1)	0.16	-0.05 (-0.1; 0.02)	0.17	0.02 (-0.04; 0.1)	0.50	-0.02 (-0.1; 0.04)	0.56
2 kHz	0.04 (-0.1; 0.2)	0.35	0.05 (-0.1; 0.02)	0.33	0.03 (-0.1; 0.1)	0.55	0.03 (-0.1; 0.1)	0.45
4 kHz	-0.02 (-0.1; 0.1)	0.64	-0.02 (-0.1; 0.1)	0.62	-0.01 (-0.1; 0.1)	0.66	0.00 (-0.1; 0.1)	0.92
SSQ-12 (baseline) for corresponding domain	0.32 (0.2; 0.5)	<0.001	0.48 (0.4; 0.6)	<0.001	0.41 (0.3; 0.6)	<0.001	0.49 (0.3; 0.6)	<0.001
Constant	2.72 (-3.3; 8.7)	0.37	-0.66 (-6.1; 4.8)	0.81	3.40 (-1.7; 8.5)	0.19	1.35 (-3.3; 6.0)	0.57

Statistical method: Multiple linear regression analysis with applied bootstrapping with 5,000 replications. Word Recognition Scores, WRS; Motivation Question One and Two from Ida Institute (Clark, 2010), Motivation Q1, Motivation Q2; Real-Ear Insertion Gain, REIG.

Statistical method: Multiple linear regression analysis with applied bootstrapping with 5,000 replications. Word Recognition Scores, WRS; Motivation Question One and Two from Ida Institute (Clark, 2010), Motivation Q1 and Motivation Q2; Real-Ear Insertion Gain, REIG, Baseline SSQ-12 score, B_SSQ-12.

Significant *p*-values marked in bold.

Patients with 10% higher WRS reported 0.4 (95%CI: 0.01; 0.1, *p =* 0.02) scale points higher SSQ-12 score per item in the space domain, and those with higher motivation for HA treatment reported a 0.2 (95%CI: 0.1; 0.4, *p =* 0.03) scale points higher SSQ-12 space score per one unit change in motivation score. WRS and motivation had similar significant effects on the total SSQ-12 score. Female HA users reported 0.7 (95%CI: 1.3; -0.2, *p =* 0.01) units lower SSQ-12 space score per item in the space domain than male HA users. These results indicate that WRS, motivation, and sex can also modestly affect SSQ-12 scores.

### 3.3 Real-ear measurements

#### 3.3.1 Differences in insertion gain deviation from NAL-NL2 target at first-fit

Table 5 shows the mean difference between the real-ear insertion gain at 65 dB SPL input level and NAL-NL2 target at 0.5, 1, 2, and 4 kHz for the six HAs at first-fit. There was no statistically significant difference in how much the measured insertion gain deviated from NAL-NL2 target gains between premium and basic HAs across the three companies (A, B, and C). However, comparing the gain deviations between premium and basic HAs within company A, B, and C revealed that there was a statistically significant difference in gain deviation from NAL-NL2 within company A at 2 kHz (mean diff: 3.7 dB; *p <* 0.001) between the premium and basic HA and for company B at 1 and 2 kHz (mean diff: 2.3 dB and 2.7 dB; *p <* 0.05) The mean difference between the insertion gains and NAL-NL2 target gains at the measured frequencies varied from 2 to 3 dB between the two levels of technology.

**TABLE 5:** Mean differences (in dB) between real-ear insertion gain at manufacturers’ first-fit and NAL-NL2 prescription target for the six hearing aids.

Frequency, kHz	Hearing aids					
	Basic A	Premium A	Basic B	Premium B	Basic C	Premium C
0.5	0.6 (2.0)	0.3 (0.9)	0.8 (1.9)	0.1 (2.7)	0.1 (1.5)	0.3 (1.7)
1.0	−3.2 (2.7)	−3.3 (2.1)	2.1 (4.1)*	−0.2 (3.6)	−2.6 (3.8)	−3.1 (3.7)
2.0	−5.7 (3.3)**	−2.0 (3.6)	−0.9 (4.6)*	−3.6 (4.4)	−2.8 (3.6)	−1.3 (4.3)
4.0	−11.4 (4.0)	−9.3 (4.4)	−8.4 (5.1)	−9.9 (4.4)	−6.9 (4.5)	−6.1 (5.1)

Data for left and right ears were averaged for each patient (n = 162). Standard deviations are given in parentheses. *significance at *p* < 0.05, **significance at *p* < 0.001when comparing data for each pair of premium and basic hearing aid.

#### 3.3.2 Differences in real-ear insertion gain at first-fit

**FIGURE 3:**
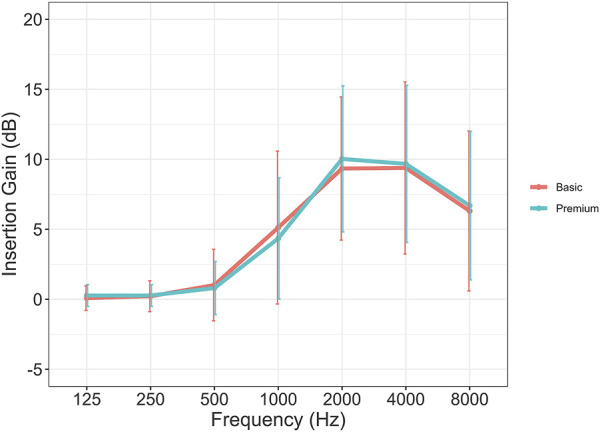
Average insertion gain at 65 dB SPL input level by level of device technology (n = 162). Error bars show 95%CI.

**FIGURE 4:**
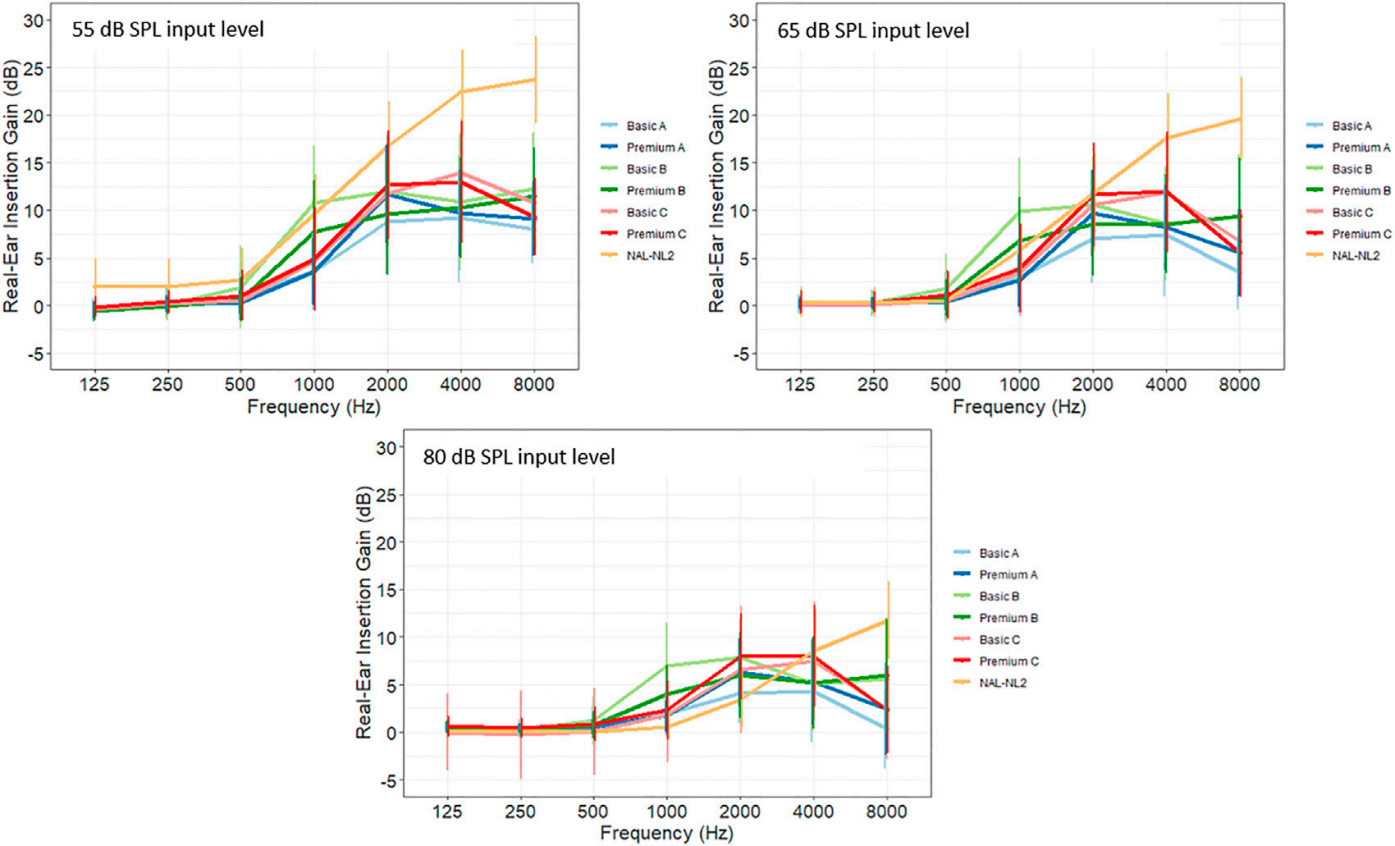
Average insertion gain at 55, 65, and 80 dB SPL input level by level of device technology and companies **(A-C)**. NAL-NL2 is shown as a reference target (n = 162).

Figure 3 depicts the mean insertion gain from 125 to 8,000 Hz for the first-fit of premium and basic HAs at 65 dB SPL. Data are given for the average of the left and right ears and NAL-NL2 is included as a reference target. The figure demonstrates the average prescribed gain is highly similar for the three premium and basic HAs used in the current study. However, looking at the gain levels for premium and basic HAs within each company (A, B, and C) at the three input levels (55, 65, and 80 dB SPL) shown in Figure 4, a difference in the prescribed gain between premium and basic HAs can be observed. The largest difference between premium and basic HA within each company is observed at 1 kHz (company B) and 2 kHz (company A and C). The absolute value of the (mean) difference between premium and basic HA was calculated to be approximately 3 dB gain at 1 kHz in company B and 2 dB gain at 2 kHz in company A and C, which are rather small gain differences.

## 4 Discussion

One of the main findings from the current study was that presbycusis patients without previous HA experience using premium HAs reported better hearing abilities in terms of speech and qualities of hearing compared to those using basic HAs using the SSQ-12 as outcome measure. These results suggest that patients with symmetric presbycusis might benefit from premium technologies with regards to improved hearing abilities. Using the IOI-HA, showed no significant difference in reported HA effectiveness between the two levels of HA technology. In total, four out of seven outcome variables failed to show a significant difference between premium and basic HAs, and with larger samples sizes, even small effects can yield statistical significance. Hence, it is important to acknowledge the limited clinical relevance of the results. Also, given the rather small differences in SSQ-12 scores between the premium- and basic-feature HA users (mean differences ranged from 0.6 to 0.8 scale points), it is questionable if these observed differences would be relevant to daily life. Although minimal clinically important differences have not been established for the SSQ-12, previous research has used one scale point of change to demonstrate a clinical significant change between assessments in SSQ (Noble & Gatehouse, 2006; Lenarz et al., 2017)). However, Lenarz et al. (2017) looked at the change in SSQ at an individual level (within-effect) while our study investigated mean group differences (between-effect), and therefore the clinically relevant difference in SSQ might be less than one scale point. In Plyler et al. (2021), the observed differences in SSQ between the two device technology levels were smaller (0.2–0.5) compared to our study and was not found statistically significant.

In contrast, previous studies did not provide evidence to suggest that premium HAs yield better self-reported real-world outcomes or laboratory outcomes (Cox et al., 2014; 2016; Johnson et al., 2016; 2017; Plyler et al., 2021; Saleh et al., 2021). Although Wu et al. (2019) found that premium HAs provided better speech understanding and sound localization in the laboratory, this improvement was not transferred to the real-world setting. Cox et al. (2014) combined three sets of questionnaire data (SSQ-B, DOSO, and APHAB) to provide one single benefit score which may have affected the sensitivity of the original scales and concealed any observed differences in the reported benefit. One possible explanation for the different findings is that in the present study, patients used either a premium or a basic device, whereas the participants in previous studies tried both levels of technology. This could have biased their experience by comparing between different HA models, but it also could have helped them to decide which one they preferred, and the fact that they were all blinded to the model name and technology level limits this bias. Experienced users commonly exhibit a more severe hearing loss, which is why the inclusion of both first-time and experienced users in the previous studies might have contributed to a larger variation in hearing thresholds that could influence differences in reported hearing abilities related to the level of device technology. The mean audiograms in Cox et al. (2014; 2016) shows PTA-4 thresholds above 70 dB HL and SDs above 20 dB HL, whereas in our study that only included first-time users, the PTA-4 ranged from 15–64 dB HL and IQR from 10 to 12.5 dB HL (Table 3).

Another important difference between previous studies and the current study is that the HA technology used in the previous studies was at least one developmental epoch older than the current study, and the technology advances could therefore be expected to be more evident in the premium devices used in the current study. Hence, it is noticeable, that even with greater contrast in technology, the IOI-HA total score along with the Factor 1 and Factor 2 scores failed to show a significant difference between premium and basic level HAs. This suggests that the perceived effectiveness using premium and basic technologies were similar. However, as the IOI-HA addresses the overall effectiveness with HAs and the SSQ addresses the hearing (dis)abilities in different specific listening situations, we speculate if the IOI-HA items were not specific enough to detect perceived differences between the two technology levels. Thus, the SSQ-12 appears to be a more sensitive outcome measure to uncover small differences in technology levels or feature settings.

One explanation for premium HAs yielded better perceived hearing abilities in the SSQ speech domain could be that the more advanced features in the premium HAs can be better at distinguishing speech signals from noise than basic-feature HAs. The high-frequency boost feature and the speech-enhancer feature in premium HAs (Table 1) could contribute to better reported hearing abilities by amplifying speech signals and improving speech understanding in noisy environments. Although premium users reported better hearing abilities, only small outcome differences were observed between premium and basic devices. It could be that the listening environments in their daily lives were mostly quiet, so that the directional microphones or noise reduction algorithms were not activated most of the time, and because the advanced features in premium devices are only beneficial in more demanding listening environments, the benefits of using premium technology may go unnoticed (Wu et al., 2019). The occupational status revealed that the majority of HA users in the two study groups were retired (81%–84%, respectively), which could indicate less demanding sound environments in their daily life. This is line with results from Wu et al. (2019) that found only 10.9% of the self-reports were conducted in noise, which led the authors to the same conclusion. Nevertheless, because the logging data from the six HAs differ significantly across brands, except from use-time that was extracted, the comparability of these data is limited. The slightly better reported SSQ outcomes using premium HAs could also be related to non-signal-processing factors, such as connectivity and having access to smartphone user-controlled settings. Saleh et al. (2021) investigated drivers of user preference between premium and entry-level HAs using group concept mapping approach and found that these non-signal-processing factors significantly influenced the preference of premium HAs. This underlines the importance of including non-audiological features, such a connectivity, in modern HAs and could have a significant impact on the reported outcomes using premium HAs.

Female HA users reported significantly poorer hearing abilities than men in the space domain related to questions on directional and distance hearing. Previous studies have shown that females reported poorer outcomes than men using the IOI-HA which was related to Factor 2 scores (communication with others) (Arlinger, Nordqvist and Öberg, 2017; Houmøller et al., 2021), and they suggested this might be due to women being more socially active than men and consequently exhibit higher expectations towards the HAs.

Insertion gain levels at first-fit measured with REM showed that premium HAs prescribed more high-frequency gain than basic HAs for company A, but the opposite was found for company B (Figure 4). The gain differences within companies A, B, and C might reflect different fitting strategies, and the change in fitting strategy for company B, reported in Smeds et al. (2016), could explain why more gain is prescribed at 1 kHz for the basic-feature HA compared to the premium HA (seen in Table 5). Nevertheless, the insertion gain differences did not explain the small differences in reported SSQ outcomes as shown in Table 4, but it was striking to find that different gain levels are prescribed for highly similar types of presbycusis hearing losses. This finding is consistent with other studies that also showed extensive differences in amplification characteristics between different manufacturers’ proprietary fitting algorithms for the same type of hearing loss (Keidser, Brew and Peck, 2003; Sanders et al., 2015). A substantial gain deviation from NAL-NL2 target was found in the high-frequencies (at 4 kHz) for all six HAs (Figure 4; Table 5) and could be explained by the intervention in this study followed standard practice in Denmark in which the adjustments at the fitting session are conducted in a non-systematic way. It is common practice in clinic to initially lower the gain in the high-frequencies, especially at 4 kHz, as part of an acclimatization. Thus, the proprietary initial fit might have prescribed more high-frequency gain than measured at the follow-up, but it is important to remember that all HAs were fitted using the proprietary fitting algorithm, which differs from the NAL-NL2 target. Even though Bell (2009) states that the quality of match to target is limited by the number of channels in the HA, and that the premium devices used in this study did include more compression channels, modern HAs–both premium and basic–in general have many compression channels, which are expected to allow for an accurate fit. The results from this study confirm this statement.

Although most previous studies did not find real-world differences between premium and basic devices, individual characteristics such as noise acceptance and listening demands along with satisfaction in large groups showed to have an impact on the perceived benefit of different HA technologies (Plyler et al., 2021). Those in more demanding listening environments reported significant improvement with premium HAs compared to those in less demanding sound environments. Also, the preference of using premium technology was found to be a factor in Cox et al. (2016), Plyler et al. (2021), and Saleh et al. (2021). These results indicate that candidacy for premium technologies and auditory ecology are highly relevant aspects that should be included in the HA provision process to improve current clinical procedures.

One of the aims of the BEAR project is to evaluate and improve the current hearing rehabilitation, including the HA prescription. Thus, to improve the clinical procedures in the HA provision process and achieve a personalized HA fitting, it is important that clinicians perform a thorough candidacy evaluation and consider the patients’ individual listening goals and auditory ecology, including the listening needs in their everyday life acoustic environments (Gatehouse et al., 1999; Jensen & Nielsen, 2005).

This study aimed to investigate if arguments for prescribing a more costly premium HA for older adults with presbycusis could be found, thereby providing clinical guidance in HA prescription. We found only small differences on a subset of the self-reported outcomes between premium and basic level HAs. When HA cost is not covered, studies have shown that patients perform a cost-benefit analysis to decide if the premium HAs provide sufficient additional value to justify the extra cost. In this case, the willingness and ability to pay are important factors during the decision process. In countries were the HAs are free of charge as part of the public healthcare system, it is up to the clinician to decide if premium technologies are worth the extra cost, and therefore, the decision is based on the individual patient-clinician relationship. A further analysis of the cost-effectiveness of the HA treatment would be interesting but was beyond the scope of the present study.

### 4.1 Strengths and limitations

The main strength of the current study lies in its study design, which allows for a highly homogenous study population including only patients with symmetric presbycusis. This minimized the variation in patient demographics excludes the risk of different types of hearing loss to affect the results and is a clear advantage compared to other similar studies. In contrast to previous studies, the current study only included first-time HA users to exclude bias towards a certain HA model that would have been a potential risk using experienced users. In addition, this study investigated a larger study population, including 190 patients compared to 24, 25, or 45 subjects, respectively, included in previous studies (Cox, 2014; Cox, 2016; Johnson, 2016; Johnson, 2017; Plyler et al., 2021). However, it is important to note that the pathology behind the presbycusis hearing loss can be different for the patient cohort in the current study which yields different audiometric phenotypes according to Dubno et al. (2013). Thus, differences in phenotype might affect the reported hearing ability. The fact that the HAs used in the current study were free of charge can be considered as a strength, as this removes the economic bias that is found to be an important factor for perceived HA benefit. Further, it was also a strength that the patients in the current study were treated as a part of a much larger study group (the BEAR group), and therefore no special attention was given towards the fitting of these patients. The researchers were not actively involved in collecting data before the follow-up, and they were blinded towards the level of technology for the analysis, hence, blinding was carefully controlled to limit researcher bias.

An important limitation to consider is that the outcome measures used in this study reflect the patients’ perspective and might not be sensitive to the actual differences observed in their daily lives. Using self-reported measures such as the SSQ-12, we do not know if the addressed listening conditions were relevant or important to each HA user, or whether other relevant or important conditions were not addressed. A sentence-based test like HINT (Nielsen and Dau, 2011) or other Matrix tests (Kollmeier and Wesselkamp, 1997; Wagener, 2004; Gazia et al., 2022) could have been a more accurate measure to reveal the differences between premium and basic HA technology. The lack of user-environment logging data is a limitation of the study that could have provided insights to potential differences in their daily life sound environments. Another important aspect to consider is that the “wow-effect” among first-time users may dilute the perceived differences between device technologies and therefore less likely to detect differences between premium and basic technologies. However, as this study compares two groups of first-time users using only one technology level, this risk limited. Although patients reported no prior HA experience, they might have been previously fitted with HAs from a private vendor for a shorter trial period, but this was not included in the referral letter from the ENTs. The HA fitting in this study followed the current protocol in Denmark in which the adjustment of HAs are managed in a non-systematic way. We acknowledge that it might be a potential limitation of the current study, but we tried to control for this by including mean gain deviations in the regression analysis. The maximum power output (MPO) of each individual fitting was not registered, so we are unaware of potential differences in MPO between the premium and basic HA models. The counselling part was also non-systematic which might bias the results, but due to the randomized trial design, this potential bias should be equally representative across the two groups. At the time of inclusion, it was not known if patients were still employed full-time, or if they had unusually high leisure activity levels, so that the randomization of HA technology could not be based on these factors. However, as reported in Table 1, the vast majority of patients were retired. One could speculate that the results would be affected by including more patients in full-time employment because it might increase the need for more advanced feature settings in their HAs due to more difficult listening environments or higher listening demands. Patients’ personalized listening goals were not addressed as suggested in the best-practice guidelines, which are considered important for the perception of the HA fitting. Also, the fact that patients were not completely blinded in regards to the selected type of HA could have introduced some bias into the results. If patients knew they were fitted with a more advanced HA model, it might have affected the reported outcome measures. Finally, the generalizability of the current study is limited to community-dwelling older adults with presbycusis and without previous HA experience.

## 5 Conclusion

The current study aimed to provide clinical guidance in HA prescription, to improve current clinical procedures for older adults with presbycusis. Premium HAs yielded slightly better self-reported hearing abilities in older adults with symmetrical presbycusis without previous HA experience, but this does not necessarily apply to other types of hearing loss, and the clinical relevance of the results is limited. Hence, there is limited evidence to support the choice of more costly premium technologies, and clinicians should therefore be careful not simply to conclude that more advanced technologies always produce a better outcome. Differences in real-ear insertion gain at first-fit did not explain the differences in reported outcome. Hearing care providers should continue to insist on evidence to support the choice of more costly premium technologies and include aspects as candidacy and auditory ecology in the HA provision to achieve a more personalized fitting and improve rehabilitation outcome.

## Data Availability

The raw data supporting the conclusion of this article will be made available by the authors, without undue reservation.
